# SARS-CoV-2 pre-exposure prophylaxis: A potential COVID-19 preventive strategy for high-risk populations, including healthcare workers, immunodeficient individuals, and poor vaccine responders

**DOI:** 10.3389/fpubh.2022.945448

**Published:** 2022-08-08

**Authors:** Jing Ouyang, Silvere D. Zaongo, Vijay Harypursat, Xiaofang Li, Jean-Pierre Routy, Yaokai Chen

**Affiliations:** ^1^Clinical Research Center, Chongqing Public Health Medical Center, Chongqing, China; ^2^Infectious Diseases and Immunity in Global Health Program, Research Institute, McGill University Health Centre, Montréal, QC, Canada; ^3^Chronic Viral Illness Service, McGill University Health Centre, Montréal, QC, Canada; ^4^Division of Hematology, McGill University Health Centre, Montréal, QC, Canada; ^5^Division of Infectious Diseases, Chongqing Public Health Medical Center, Chongqing, China

**Keywords:** COVID-19, pre-exposure prophylaxis (PrEP), high-risk population, molnupiravir, remdesivir

## Abstract

The unprecedented worldwide spread of SARS-CoV-2 has imposed severe challenges on global health care systems. The roll-out and widespread administration of COVID-19 vaccines has been deemed a major milestone in the race to restrict the severity of the infection. Vaccines have as yet not entirely suppressed the relentless progression of the pandemic, due mainly to the emergence of new virus variants, and also secondary to the waning of protective antibody titers over time. Encouragingly, an increasing number of antiviral drugs, such as remdesivir and the newly developed drug combination, Paxlovid^®^ (nirmatrelvir/ritonavir), as well as molnupiravir, have shown significant benefits for COVID-19 patient outcomes. Pre-exposure prophylaxis (PrEP) has been proven to be an effective preventive strategy in high-risk uninfected people exposed to HIV. Building on knowledge from what is already known about the use of PrEP for HIV disease, and from recently gleaned knowledge of antivirals used against COVID-19, we propose that SARS-CoV-2 PrEP, using specific antiviral and adjuvant drugs against SARS-CoV-2, may represent a novel preventive strategy for high-risk populations, including healthcare workers, immunodeficient individuals, and poor vaccine responders. Herein, we critically review the risk factors for severe COVID-19 and discuss PrEP strategies against SARS-CoV-2. In addition, we outline details of candidate anti-SARS-CoV-2 PrEP drugs, thus creating a framework with respect to the development of alternative and/or complementary strategies to prevent COVID-19, and contributing to the global armamentarium that has been developed to limit SARS-CoV-2 infection, severity, and transmission.

## Introduction

Coronavirus disease 2019 (COVID-19), caused by severe acute respiratory syndrome coronavirus 2 (SARS-CoV-2), has, over the past 2 years, resulted in the death of millions of people globally (1). Moreover, COVID-19 has dramatically affected and altered the lives and livelihoods of people in every corner of the world due to its effects on local, regional, and global health care systems, economies, environments, and geopolitical posturing. During this period, various reactive, adaptive, and defensive coping strategies employed by government authorities, such as regional lockdowns, the use of SARS-CoV-2 vaccines, and antiviral drugs, have been implemented, which have influenced the evolution of the pandemic.

The roll-out of several different COVID-19 vaccines by pharmaceutical companies has been considered a major milestone in the global medical effort to prevent populations from developing severe disease from SARS-CoV-2 infection. With respect to the protective effects of these vaccines, data indicates that cellular immunity induced by COVID-19 vaccines protected against severe infection, even against new SARS-CoV-2 variants (2–4). Keeton et al. reported that the T-cell responses induced by COVID-19 vaccination or previous SARS-CoV-2 infection are cross-reactive with the Omicron variant of SARS-CoV-2, despite extensive mutation and reduced susceptibility to neutralizing antibodies of the Omicron variant (2). Nonetheless, the much anticipated protective effects of COVID-19 vaccines have been found to be limited and transient for two main reasons: (1) Protective and neutralizing antibody levels wane after a few months post vaccination, and (2) The SARS-CoV-2 virus undergoes active genomic mutation, rendering some COVID-19 vaccines functionally obsolete even before they have been utilized at a population level (5–9). One multicenter prospective study, conducted by Favresse et al. observed that vaccine-associated antibody titers decline post-vaccination with the BNT162b2 mRNA COVID-19 vaccine (Pfizer, BioNTech) (Comirnaty^®^), with an estimated antibody half-life of 55 and 80 days for seropositive and seronegative subjects, respectively (6). It has also been reported that vaccine-associated antibody titers achieve peak levels at 1 month after the second dose of the BNT162b2 mRNA COVID-19 vaccine, and subsequently rapidly decrease over time (9). Moreover, newer variants of SARS-CoV-2 may be evolving into virions capable of vaccine-breakthrough, containing several antibody-resistant mutations, such as the Omicron SARS-CoV-2 variant, which is a heavily-mutated virus variant, and is classified as a variant of concern (VOC) by the World Health Organization (WHO) (10). Based on three-dimensional (3D) renderings of its antibody-receptor-binding domain (RBD) complex structures, Chen et al. has claimed that the Omicron variant has an 88% likelihood of evading antibodies generated by current vaccines (11). The Omicron (B.1.1.529) variant could also increase the risk of SARS-CoV-2 reinfection, which is associated with immune evasion (12). Similarly, Hoffmann et al. have reported that the Omicron variant evades neutralization by antibodies from vaccinated individuals with up to 44-fold higher efficiency than the Delta variant (13). Also, microbial dysbiosis, gut barrier integrity loss, and/or microbial translocation are also thought to be involved in the milieu of COVID-19 disease and poor host immune responses secondary to vaccination (14, 15). This indicates that alternative strategies to vaccination to combat COVID-19, geared toward supplementing and consolidating the existing defensive arsenal, are warranted.

Pre-exposure prophylaxis (PrEP) refers to the utilization of medication/drugs before risk exposure, in order to prevent disease acquisition and transmission, for people at high risk to be infected. PrEP has typically referred to the prevention of HIV infection using specific antiviral agents by a person at risk of HIV acquisition. It has been deemed a cornerstone for HIV prevention. Convergent evidence from prospective clinical trials has demonstrated the efficacy of HIV PrEP in reducing the risk of HIV acquisition, which is known to be up to 98% effective when adherence to treatment is optimal (16–21). Several HIV PrEP drugs and drug combinations have been recommended by the WHO, and these have been employed for use in people at high risk of HIV infection, as a part of the combination HIV prevention approach (22).

With respect to SARS-CoV-2, several antiviral agents have now been investigated and developed that show inhibitory effects against SARS-CoV-2 both *in vitro* and *in vivo*, including remdesivir, molnupiravir, and Paxlovid^®^ (nirmatrelvir/ritonavir). In this review, we propose that PrEP using the preceding antiviral drugs, as well as other potentially effective anti-SARS-CoV-2 agents, might be considered to prevent SARS-CoV-2 acquisition in high-risk populations, inspired by the unparalleled success of PrEP in preventing the acquisition of HIV.

Herein, we critically review the risk factors for severe COVID-19, discuss potentially viable SARS-CoV-2 PrEP strategies, as well as their limitations in targeted populations, thus paving the way for the development of an alternative or complementary strategy to prevent SARS-CoV-2 infection and secondary transmission.

## High risk population for COVID-19

The population that has a substantially higher probability to closely interact with SARS-CoV-2-infected individuals, and who would be considered as the group with the highest risk of exposure to SARS-CoV-2 infection, would be healthcare workers. A significantly large number of healthcare workers have already been infected by SARS-CoV-2, and some of these infected individuals experienced poor outcomes, especially at the early stages of the pandemic (23–25). In October 2021, the WHO estimated that, globally, between 80,000 and 180,000 healthcare workers died due to COVID-19 between January 2020 and May 2021 (26–28). Aside from close contact, there are multiple other risk factors, such as older age, presence of comorbidities, environmental factors, and poor vaccine response, which may facilitate SARS-CoV-2 infection and produce unfavorable outcomes, including long-COVID-19.

### Demographic factors

Convergent investigational observations indicate that susceptibility to and disease severity of COVID-19 are associated with older age, male gender, and ethnicity (29–31). Data from the early stages of the pandemic in US indicates that case-fatality rates increase with age, with <1% of deaths among people aged ≤ 54 years of age, 1.4–4.9% among people aged 55–74 years of age, 4.3–10.5% among people aged 75–84 years, and 10.4–27.3% among people aged ≥ 85 years old (29). Moreover, one global meta-analysis conducted in 2021, which included 59 studies and 36,470 patients, observed that males and the older population have a materially higher risk for SARS-CoV-2 infection, severe disease, and mortality (30). In concordance with these findings, another meta-analysis, which included 14 studies, 29,909 SARS-CoV-2-infected patients, and 1,445 cases of death, indicated that older age (≥65 years old) and male gender were associated with a greater risk of death from COVID-19 infection, with a pooled odds ratio (OR) of 4.59 [95% confidence interval (CI) = 2.61–8.04, ≥65 vs. <65 years old], and 1.50 (95% CI = 1.06–2.12, male vs. female) (31). Older age is unavoidably associated with various other comorbidities, poor immunity, and increased levels of circulating pro-inflammatory cytokines (32). Additional factors, such as differences in levels of circulating sex hormones between men and women, levels of ACE2 enzymes and receptors, the presence of the transmembrane serine protease 2 (TMPRSS2) enzyme, and lifestyle factors such as smoking, may also contribute to variable risks of severity and mortality of COVID-19 in afflicted persons (32, 33).

Generally, it has been considered that non-Caucasian races are associated with increased risk of SARS-CoV-2 infection, disease severity, and mortality, compared to people of Caucasian ancestry (34–36). One meta-analysis, which included 18,728,893 patients from 50 studies, observed that individuals of black and Asian ethnicity are at increased risk of SARS-CoV-2 infection compared to Caucasian individuals, and that Asian individuals are at higher risk of ICU admission and death (34). However, after adjusting for comorbidities, another meta-analysis reported that racial discrepancies observed in risk of SARS-CoV-2 infection rates may actually be attributable to higher comorbidity prevalence in certain racial groups (37).

ABO blood groups have also been found to be associated with COVID-19 susceptibility, severity, and mortality (38–43). Group A individuals showed an increased risk of becoming infected by SARS-CoV-2, compared to group O (39–43). With respect to severity and mortality, Muñiz-Diaz et al. reported that specific ABO blood groups were also seen to represent important risk factors for development of COVID-19, with the risk in Group A individuals being significantly higher than that in Group O individuals (38).

### Comorbidities

A large volume of published literature has reported that various comorbidities may predispose patients with COVID-19 to an unfavorable outcome, and a higher risk of death (44–51). Hernández-Garduño, after analyzing the data of 32,583 patients, showed that the presence of either obesity, diabetes, or hypertension were strong predictors for both the acquisition of SARS-CoV-2 infection and the development of severe disease (50). COVID-19 clinical guidance issued by The American College of Cardiology indicates that case fatality rates for comorbid COVID-19 patients are substantially higher than the average population, i.e., case fatality rates for comorbid cardiovascular disease (CVD) being 10.5%, diabetes (7.3%), chronic respiratory disease (6.3%), hypertension (6.0%), and cancer (5.6%) (51).

The United States (US) Centers for Disease Control and Prevention (CDC) has released a comorbid medical condition list for COVID-19, and has issued advice stating that having one of the listed conditions may make a person more likely to become severely ill from COVID-19 (52). This updated list includes cancer, chronic kidney disease, chronic liver disease, chronic lung diseases, dementia or other neurological conditions, diabetes, etc. (52). Similarly, several meta-analyses have now confirmed that the listed conditions do indeed predispose individuals to severe illness (53–57). For instance, Thakur et al. published a meta-analysis, which included 120 studies and 125 446 patients, which observed that the most prevalent COVID-19 comorbidities were hypertension (32%), obesity (25%), diabetes (18%), and cardiovascular disease (16%), while patients having renal comorbidities had the highest severity and mortality rates (53).

### Environment

Accumulating evidence has shown that environmental and climatic factors have a significant effect on COVID-19 transmission and mortality. These factors include population density, temperature, ozone levels, sulfur dioxide levels, humidity, wind speed, and rainfall levels (58–65).

Yin et al. analyzed the data of cities in China and in the USA, and observed that a higher population density was associated with a higher percentage of morbidity related to COVID-19, indicating the importance of social distancing and travel/movement restrictions for the prevention of COVID-19 transmission (65). Sobral et al. investigated the association between climatic conditions and global SARS-CoV-2 transmission, and found that, aside from prevailing average temperature levels, countries with higher rainfall measurements showed an increase in COVID-19 transmission, with each average inch/day of rainfall equating to an additional 56.01 newly-identified COVID-19 cases/day (62). Generally, higher population density, air pollution, rainfall, and wind speed, as opposed to lower temperature, humidity and sulfur dioxide levels are associated with higher COVID-19 infection and severity rates (58–65).

### Vaccine responses

The development of protective immunity after COVID-19 vaccination relies on long-term B- and T-cell memory responses to SARS-CoV-2 (66, 67). Immunosuppressed patients, such as those with immunodeficiency, organ transplant recipients, untreated HIV-infected patients, and cancer patients who have B- or T-cell deficiency are more likely to develop severe COVID-19 (68, 69). Goubet et al. reported that the lymphopenia was associated with prolonged SARS-CoV-2 RNA virus shedding and poor prognosis in cancer patients (70). In SARS-CoV-2 susceptible individuals, Fahrner et al. (67) observed a specific deficit in the TH1/Tc1 response against the receptor binding domain of the spike protein (S1-RBD), and vaccine-induced S1-RBD TH1 immunity is reduced in hematological malignancies. Fernandez Salinas et al. (71) reported that only 33% of patients with common variable immunodeficiency (CVID) showed an antibody response to the COVID-19 vaccine; moreover, CVID could not generate RBD-specific MBCs even after two vaccine doses, compared to healthy vaccinated individuals. Amodio et al. (69) reported that five patients with inborn errors of immunity (IEI) did not mount any cellular response, as is usually observed in healthy individuals, following the BNT162b2 mRNA COVID-19 vaccine, and one of these patients was also found to not be able to mount any humoral response. Thus, for these immunosuppressed populations, alternative or complementary strategies would be lifesaving.

## Lessons from the PrEP strategy for HIV

Evidence indicates that HIV PrEP is extremely effective (up to 98% effective) in reducing the risk of HIV acquisition when adherence to PrEP is optimal (16–20). PrEP has been broadly utilized to prevent HIV spread for populations who have a higher risk of acquiring HIV, such as sex workers, people who engage in recreational injection drug use, and those who practice unprotected receptive anal intercourse. Two combinations of oral antiretroviral drugs have been approved and used for HIV PrEP, including the combination of tenofovir disoproxil and emtricitabine (Truvada^®^), and tenofovir alafenamide and emtricitabine (Descovy^®^). Additionally, in December 2021, the US Food and Drug Administration (FDA) approved the first injectable preparation (cabotegravir extended-release injectable suspension) for HIV PrEP, which is believed to greatly improve medication compliance as it is administered only every 2 months.

Concerns regarding long-term drug safety, cost, development of drug resistance, and risk compensation of HIV PrEP present ongoing challenges. Some adverse effects have been observed; however, cumulative evidence has revealed that HIV PrEP has an overall satisfactory safety profile (72). The main adverse effects are usually mild to moderate nausea, vomiting, and diarrhea. Kidney and liver toxicity are rare; however, regular monitoring of renal and liver functions are required (73). Other concerns exist, including cost, development of drug resistance, and risk compensation. One meta-analysis that included 18 studies and 19,491 participants demonstrated that PrEP was highly effective across populations, presented few adverse events and instances of drug resistance, and had no significant association with changes in sexual risk behavior (21). Overall, current evidence indicates that the benefit-risk profiles of available HIV PrEP regimes are strongly favorable for the targeted population at high risk of infection by HIV.

## Consideration for PrEP for COVID-19

**Figure 1:**
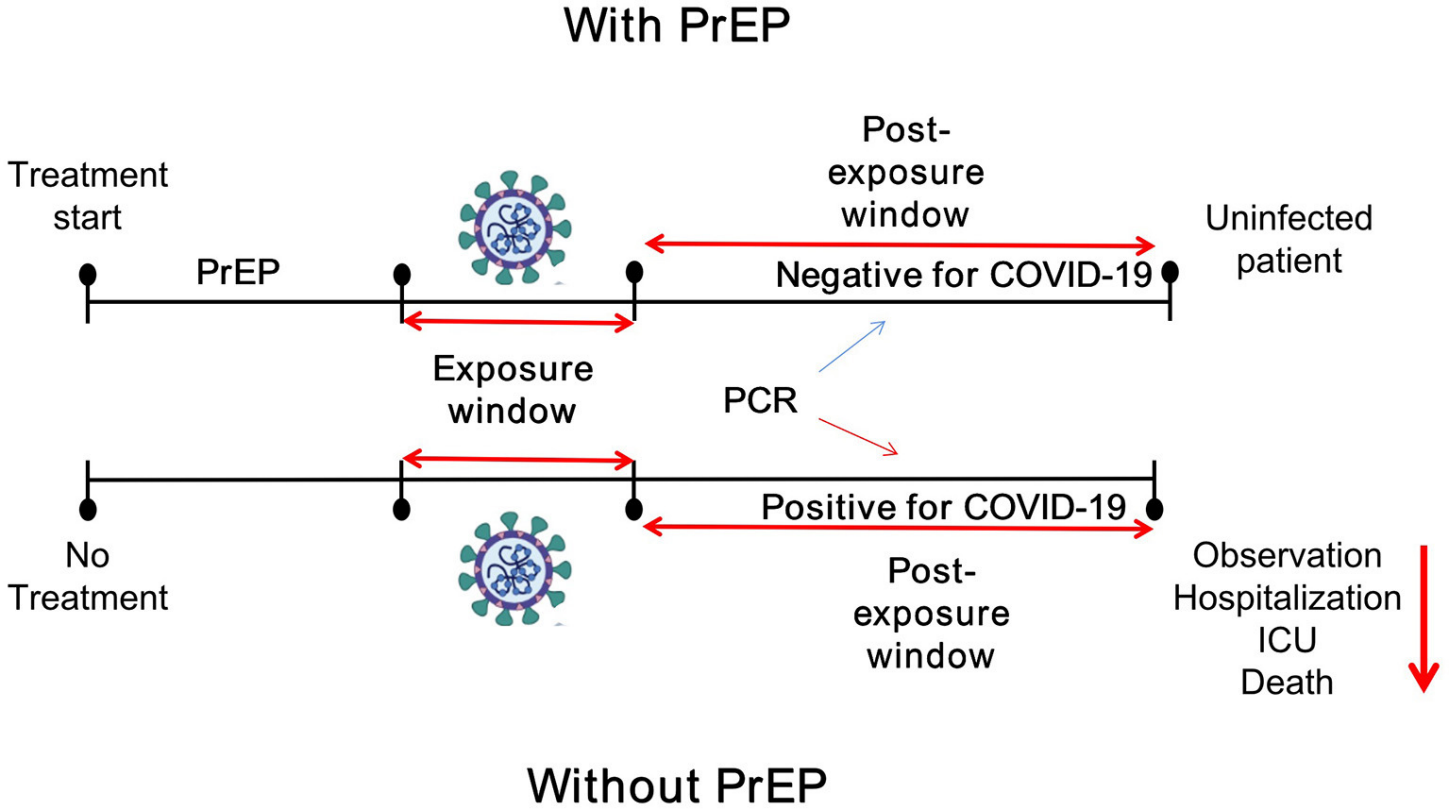
Comparison of SARS-CoV-2 PrEP strategies and post infection therapy. With an effective PrEP strategy, the patient remains negative for COVID-19 as SARS-CoV-2 cannot replicate in the patient's body. Conversely, without a PrEP strategy, any exposure to SARS-CoV-2 can result in an infection, confirmed by a positive PCR test. In the optimal scenario, the patient will develop a mild infection which will be subsequently controlled and eliminated by their immune response. ICU, intensive care unit.

**Figure 2:**
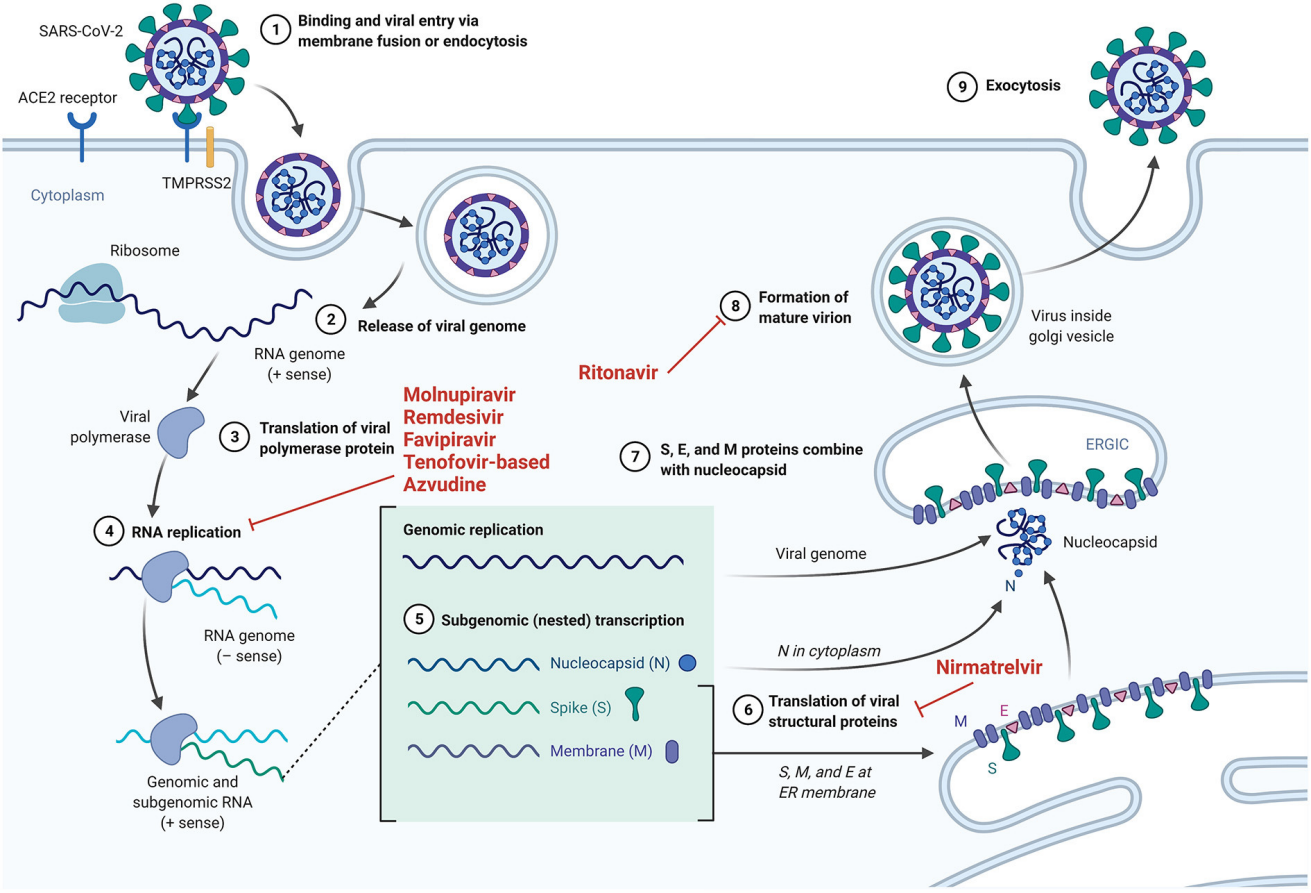
The mechanisms of action of specific drugs that could potentially serve as PrEP for SARS-CoV-2.

Drawing on knowledge gained from the use of PrEP for HIV disease, SARS-CoV-2 PrEP, using specific antiviral and adjuvant drugs against SARS-CoV-2, may represent a novel preventive strategy for COVID-19. However, unlike HIV, against which there is currently no available vaccine, SARS-CoV-2 PrEP probably will be given to individuals who have poor response to vaccination and have a high-risk of developing severe COVID-19. Moreover, SARS-CoV-2 PrEP differs from post-infection treatment, which relies on therapeutic interventions to be initiated after the patient has tested positive for COVID-19, as shown in Figure 1. We thus consider a few drugs (favoring oral administration as first choice) that could potentially be used as PrEP for SARS-CoV-2. The mechanisms of action of the drugs discussed are summarized in Figure 2.

### Molnupiravir

Molnupiravir (MK-4482) is an orally available prodrug of beta-D-N4-hydroxycytidine (NHC), a ribonucleoside that has broad antiviral activity against RNA viruses (74). Viral mutations and lethal mutagenesis results from NHC uptake by viral RNA-dependent RNA-polymerases. Molnupiravir has been found to be effective against SARS-CoV-2, as demonstrated by Zhou et al. (75) and Kabinger et al. (76). In a randomized, placebo-controlled, double-blind phase 2/3 trial, Arribas et al. observed that molnupiravir administration does not result in clinical benefit in patients hospitalized by COVID-19 (77). However, results gleaned from studies of molnupiravir administration to non-hospitalized patients indicate a much more favorable picture. Caraco et al. (78) (Phase 2 MOVe-OUT study) observed a lower incidence of hospitalization and/or death in the molnupiravir group vs. the placebo group in specific participants (especially those >60 years of age, and those with an increased risk for severe illness). The preceding research group subsequently concluded that molnupiravir administration can reduce hospitalizations and/or death in non-hospitalized patients with COVID-19. Furthermore, results from a study by Jayk Bernal et al. (79) (Phase 3 MOVe-OUT study) indicate that (i) the rate of hospitalization or death through day 29 is ~31% lower with molnupiravir than with placebo, and (ii) molnupiravir treatment is associated with greater reductions in mean viral load from baseline than placebo at days 3, 5 (end-of-treatment visit), and 10. Armed with the promising results gleaned from this study conducted in non-hospitalized COVID-19 patients, the authors concluded that early treatment with molnupiravir reduced the risk of hospitalization or death in at-risk and unvaccinated adults with COVID-19. Thus, proposing the use of molnupiravir in a pre- or post-exposure prophylaxis context seems reasonable upon reflection. Although the results from the MOVe-OUT trial indicate that molnupiravir is safe for human use, a theoretical risk with the use of molnupiravir has been postulated, as molnupiravir could possibly be processed by human host cells and could, conceivably, be incorporated into the host DNA, potentially leading to cellular mutations and cellular death (75).

### Remdesivir

Remdesivir is an antiviral nucleoside analog pro-drug which inhibits the RNA-dependent RNA polymerase non-structural protein 12 (NSP12) in SARS-CoV-2. It was originally developed to treat Ebola virus disease (80), but has shown positive outcomes when used in SARS-CoV and MERS-CoV infections *in vitro* and in preclinical *in vivo* animal models (81–83), and was also used in the treatment of the first reported case of COVID-19 in the United States of America (with no obvious adverse effects) (84). Ebola virus, SARS-CoV, MERS-CoV, and SARS-CoV-2 genomes obviously differ; however, remdesivir has a broad-spectrum of activity, and therefore has the capacity to effectively neutralize RNA polymerase, the structure and function of which is relatively similar in all RNA viruses (85, 86). Indeed, remdesivir, after a sequence of steps that is presumably initiated by esterase-mediated hydrolysis of the amino acid ester, is ultimately metabolized into the active nucleoside triphosphate analog form, which is utilized by the viral RNA-dependent RNA polymerase (RdRp) upon its diffusion into the cell. Then, utilization of that nucleoside triphosphate analog by RdRp inhibits viral replication, as it induces delayed chain termination (87, 88). It has now been established that remdesivir has potent *in vitro* activity against SARS-CoV-2 (89), and has been used and studied in several recent randomized clinical trials (90–94). Although authors arrive at differing conclusions regarding its efficacy for the treatment of hospitalized COVID-19 patients, a clinical benefit is suspected, especially when used in the early phase of the disease. Moreover, based on the manufacturer statement regarding remdesivir efficacy in preventing SARS-CoV-2 infection, the PINETREE study (NCT 04501952) showed positive effects of remdesivir on the course of COVID-19 in outpatients who were treated early, and was also shown to be safe, and well-tolerated (95). In order to circumvent the significant limitation to the use of remdesivir imposed by the requirement of intravenous administration (which may potentially limit its widespread use during the pandemic), the orally bioavailable nucleoside prodrug GS-621763, which has been shown to be metabolized into the same active nucleoside triphosphate formed by remdesivir, has now been developed, and has shown potent antiviral activity against SARS-CoV-2 in various cell models, with a similar therapeutic efficacy to intravenous remdesivir in a murine model of SARS-CoV-2 pathogenesis (96). Overall, it therefore seems reasonable to actively consider the use of remdesivir or its oral prodrug as potentially useful antiviral drugs for SARS-CoV-2 pre-exposure prophylaxis.

### Favipiravir

Favipiravir is a purine nucleoside analog which acts as a competitive inhibitor of RNA-dependent RNA polymerase (97). In other words, favipiravir has been shown to be a potent inhibitor of various different viral RNA-dependent RNA polymerases (RdRps), including in influenza A and B viruses, in several agents causing viral hemorrhagic fever, and also in SARS-CoV-2 *in vitro* (82, 97, 98). In a clinical context, favipiravir administration to COVID-19 patients has been shown to be capable of (i) reducing the window for viral clearance (from 11 to 4 days) and (ii) improving pulmonary inflammatory marker levels (91% of treated patients showed improvement vs. 62% in the control group) (99). Udwadia et al. in their randomized, comparative, open-label, multicenter, phase 3 clinical trial, have demonstrated that favipiravir administration can significantly shorten the time to clinical cure in COVID-19 patients (100). Importantly, Doi et al. have reported that early intervention with favipiravir is superior to late intervention in terms of viral clearance and time to defervescence. Favipiravir would thus be of potential benefit if administered in a SARS-CoV-2 PrEP context. Researchers conducting an ongoing clinical study in Canada are currently assessing the efficacy of favipiravir treatment over 25 days for the prevention of SARS-CoV-2 infection in residents and staff of nursing homes (among elderly, assisted-living patients, and healthcare professionals) (NCT04448119, Phase 2). Results and outcomes of this study are eagerly awaited.

### Tenofovir-based regimens

*In vitro* investigations suggest that tenofovir (i) inhibits SARS-CoV-2 RdRp, although with weaker binding than remdesivir (91, 92, 101, 102) and (ii) possesses immunomodulatory effects as it demonstrates the ability to decrease both interleukin (IL)-8 and IL-10 production (103), which are both known to favor COVID-19 severity (104, 105). In addition, observations made in people living with HIV (PLWH) indicate that there is a little evidence that HIV infection increases COVID-19 risk in settings with good access to tenofovir-based antiretroviral therapy (ART) (106). The preceding intriguing observation initiated considered ruminations with respect to the potential activity of tenofovir disoproxil fumarate (TDF, now used worldwide for HIV treatment and HIV pre-exposure prophylaxis) against SARS-CoV-2. Indeed, del Amo et al. (106), in a Spanish cohort study of 77 590 PLWH taking ART, reported that the incidence (per 10,000 persons) of COVID-19 diagnosis among patients taking TDF/FTC was 16.9 [95% confidence interval (CI), 10.5–25.9], compared to 41.7 in the general population. Furthermore, a study by Boulle et al. (107) found that PLWH taking TDF/FTC experience 59% lower mortality from COVID-19 than those taking abacavir or zidovudine (aHR, 0.41; 95% CI, 0.21–0.78). Similarly, a third cohort study by Ayerdi et al. (108) has demonstrated that HIV PrEP (tenofovir/emtricitabine**)** users who tested positive for SARS-CoV-2 antibodies showed higher rates of asymptomatic infection, although the difference in asymptomatic rates of SARS-CoV-2 infection was not statistically significant (42.7 vs. 21.7% for non-PrEP users; *p* = 0.07). In light of the tenofovir-based treatment safety profile and its putative anti-SARS-CoV-2 effects, the tenofovir/emtricitabine combination (the combination present in DESCOVY^®^ and TRUVADA^®^ for example), is currently being studied as a SARS-CoV-2 prophylactic agent. As such, we can report that (i) a clinical trial assessing the efficacy of a 12 week SARS-CoV-2 prophylaxis course of the emtricitabine/tenofovir regimen (NCT04334928) in healthcare workers in Spain is ongoing, and (ii) several additional proposed studies intend to use this specific drug combination in a preventive manner for COVID-19 (examples: NCT04519125 and NCT04405271).

### Nirmatrelvir/ritonavir

Another orally administered potentially prophylactic drug is nirmatrelvir, a specific inhibitor of the SARS-CoV-2 viral 3-chymotrypsin-like cysteine (3CL) protease (109, 110). To achieve adequate drug levels, nirmatrelvir is administered together with the CYP 3A4 inhibitor, ritonavir. The role of ritonavir, well-known as a pharmacological booster, is hypothetical in Figure 2, as it is based on theoretical evidence from several researchers (111–114) showing that lopinavir and ritonavir also inhibit the coronaviral 3CL1pro protease, although coronaviruses encode a different enzymatic class of protease. Knowing that 3CL1pro protease plays an essential role in processing the polyproteins that are translated from the viral RNA, we therefore are encouraged that ritonavir could possibly also inhibit the formation of mature virions of SARS-CoV-2. In the E,PIC-HR study (NCT04960202), 5 days of therapy with nirmatrelvir/ritonavir reduced the rate of hospitalization and/or death by 88% in COVID-19 outpatients with at least one risk factor for a severe course if therapy was started early (within 5 days) after the onset of symptoms. On the 22nd of December 2021, the US FDA endorsed and authorized nirmatrelvir/ritonavir (Paxlovid^®^) use for the treatment of COVID-19 (115). However, due to the required combination with ritonavir, drug interactions may occur, especially in high risk populations (116). Further study of this drug combination may provide a clearer picture of its benefits when administered for SARS-CoV-2 pre- or post-exposure prophylaxis.

### Azvudine

Azvudine is a safe (117) nucleoside-based broad-spectrum anti-virus clinical candidate originally developed for HIV infection treatment and prevention (118, 119). As such, azvudine was approved by China FDA for AIDS treatment on July 21, 2021 (XZXK-2021-214) in view of its efficacy in treating AIDS and its favorable safety profile during the 48-week oral treatment (120). *In vitro*, azvudine has shown significant antiviral effects against HIV (121), HCV (122), human enterovirus 71 (123), and HBV (124). Furthermore, Ren and colleagues were the first to observe a potent antiviral activity against HCoV-OC43 and SARS-CoV-2, fostering speculation with respect to its anti-COVID-19 effect. Indeed, azvudine is known to inhibit viral RNA-dependent RNA polymerase (123, 125), and in a subsequent randomized, open-label, controlled clinical trial, Ren et al. have reported in 2020 that azvudine treatment may shorten the nucleic acid negative conversion time in the mild COVID-19 context (126). They therefore requested permission for investigation using a larger sample size, to confirm their findings. Recently (in December 2021), Zhang et al. (120) have demonstrated that oral administration of azvudine was able to (i) reduce the viral load in SARS-CoV-2 infected rhesus macaques and (ii) cure all COVID-19 patients in their treatment cohort (a randomized, single-arm clinical trial; *n* = 31). They observed that all study participants demonstrated 100% viral ribonucleic acid negative conversion in 3.29 days, with a 100% hospital discharge rate in 9 days, although minor and transient side-effects (dizziness and nausea) were noted in 16.12% (5/31) of patients. It is thus valid to state that the preceding findings favor the potential utilization of azvudine in future SARS-CoV-2 pre-exposure prophylaxis strategies.

## Perspectivs and challenges for PrEP for COVID-19

Formulating, investigating, and proposing preventive strategies for COVID-19, such as SARS-CoV-2 PrEP, are likely to help prevent morbidity and mortality from COVID-19 in high-risk populations. The antiviral drugs and their combinations listed in the discussion should be considered to be theoretically and hypothetically proposed strategies for SARS-CoV-2 PrEP prevention. Even though some of the proposed therapeutic methods appear to be promising, multiple challenges remain for the future development of effective SARS-CoV-2 PrEP.

Firstly, drug adverse effects or toxicity are a primary concern. For example, there are specific host DNA mutational concerns with molnupiravir use which need to be addressed (75). Remdesivir, as a lyophilized powder or injectable solution, has been associated with renal and hepatic toxicity as a consequence of the accumulation of excipient sulfobutylether-β-cyclodextrin (SBECD) (127, 128). Moreover, most drugs listed have revealed their benefits in already-infected patients, while their efficacy and safety in preventing SARS-CoV-2 infection in uninfected and/or vaccinated individuals will warrant further studies.

Poor adherence to antiretroviral therapy against HIV has been shown to be a major determinant for the emergence of drug resistance (129). There would also be concerns regarding drug resistance development for SARS-CoV-2 PrEP. Monotherapy may well avoid drug-drug interactions; however, compared with combination therapy, monotherapy is more likely to result in the emergence of drug resistance (130, 131). Immunocompromised patients are more likely to develop high intra-host viral diversity (132–134), which further emphasizes their risk of developing drug resistance following monotherapy. Thus, further investigations should evaluate the possibility of co-administration of two or more drugs to potentially reduce the possibility of development of resistance. Thus, we believe that the US FDA-authorized nirmatrelvir/ritonavir (Paxlovid^®^) is one drug combination that can possibly be contemplated as an effective PrEP candidate.

A SARS-CoV-2 PrEP strategy may help prevent morbidity and mortality from COVID-19; however, it may also encourage the easing of the very effective preventive measures that attempt to decrease the spread of the virus, such as social distancing interventions and avoidance of exposure, thus potentially increasing infection risk. During the COVID-19 pandemic, risk compensation has been associated with vaccination and face mask use (135, 136). Risk compensation may also significantly impact the benefits of a SARS-CoV-2 PrEP strategy, especially if efficacy of SARS-CoV-2 PrEP in real-life is not sufficiently high.

Moreover, prior to the implementation of a SARS-CoV-2 PrEP strategy, the specific criteria for the likelihood of acquisition of infection after exposure to SARS-CoV-2 should also be studied and clearly defined, including the required exposure time for infection to occur (since the probability of being infected would increase when the exposure time exceeds specific time thresholds), the occurrence of new epidemic cases in the family or at the workplace, the physical distance from the potential infective spreader, and the duration of infection of the potential infective spreader (suspected infection or documented infection by PCR or rapid testing). Similarly, the follow-up of users of PrEP and the criteria evaluating the outcome of PrEP for COVID-19 remain to be clarified. A polymerase chain reaction (PCR) test performed 5 days after PrEP medication in parallel with a blood test evaluating toxicity of the drug(s) is recommended.

Furthermore, the PrEP concept excludes parenteral therapy, and should be available for high-risk patients at home, preferably before potential exposure. The dosage and the duration of treatment will depend on each drug used. Cost efficacy of PrEP should be considered with particular diligence and gravity, as such a preventive COVID-19 strategy, if effective, may circumvent ICU admission (where costs are known to be prohibitive), and extended hospital stays.

**Figure 3:**
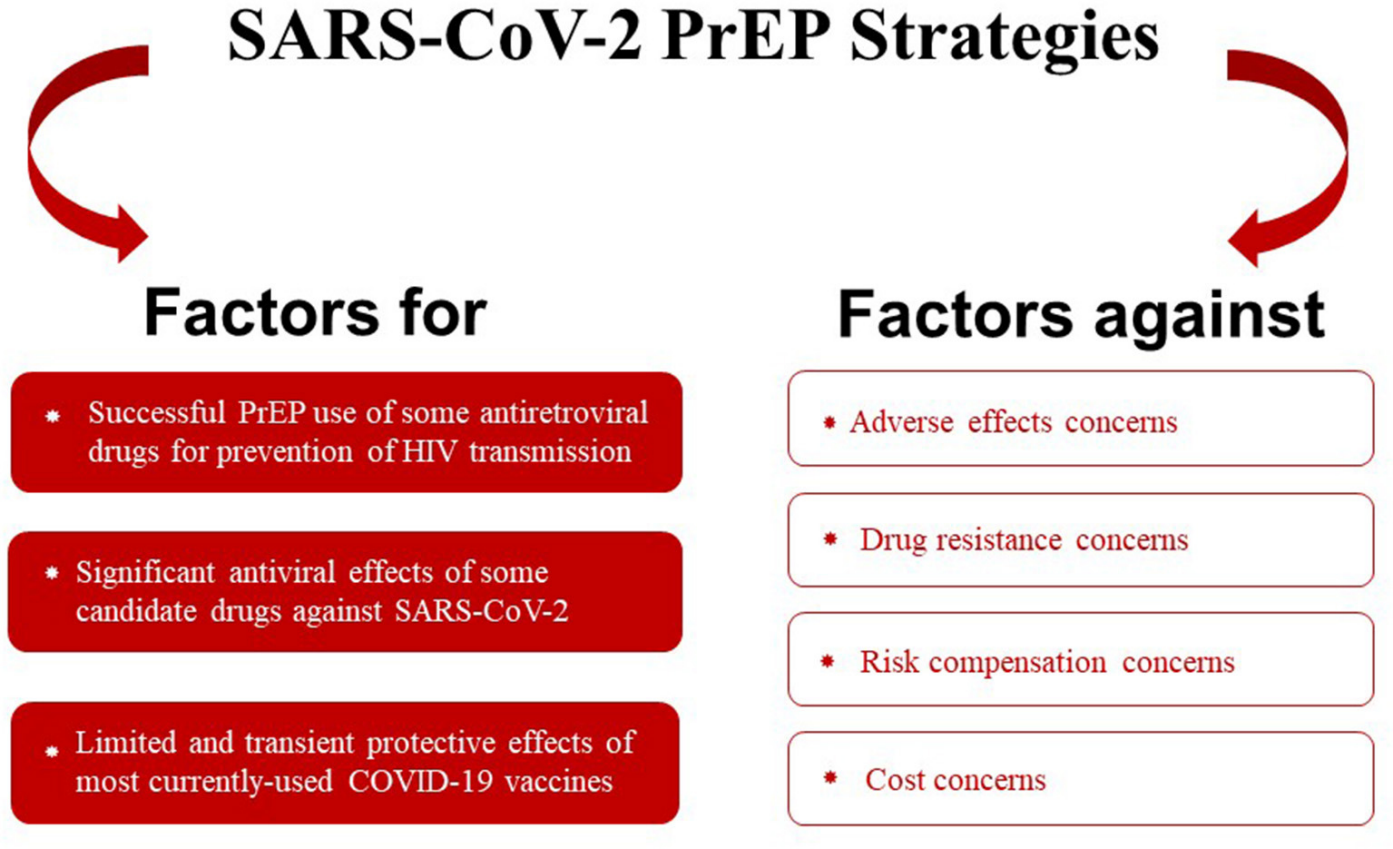
Pros and cons of SARS-CoV-2 PrEP strategies.

The PrEP for COVID-19 proposed in the preceding discussion involves the administration of the drugs listed above to only high-risk populations, particularly patients with an immunocompromised status, such as common variable immunodeficiency, lymphopenia, patients with organ transplants, and lymphoma. Additionally, the potential SARS-CoV-2 PrEP candidate drugs described herein have specific merit for use in patients who respond poorly to COVID-19 vaccination and who are more likely to develop severe COVID-19. Nevertheless, the entire SARS-CoV-2 PrEP remains a theoretical construct, and significant merits of and limitations to our proposed SARS-CoV-2 PrEP strategies exist (Figure 3). However, for selected categories of patients, PrEP for COVID-19 can likely represent a potentially viable course of action that should be carefully and impartially examined and studied.

Based on the perceived risk benefit ratio, we consider that potential SARS-CoV-2 PrEP strategies should be evaluated in the context of future SARS-CoV-2 infection waves in large populations.

Individual population health policies adopted by countries around the world have the potential to significantly challenge the PrEP strategy proposed in this article. For example, free rapid antigen testing kits are now widely available to individuals in countries such as Canada. This diagnostic test is not based the presence of SARS-CoV-2 in the test sample, but is based on specific parts of the SARS-CoV-2 virion (such as the nucleocapsid), and can thus potentially result in false positive results. The PrEP strategy that we propose is evaluated through PCR testing, which requires the presence of the virus in the test sample, and is therefore more accurate diagnostically. In Canada, based on a positive test with rapid antigen kits, pharmacists can initiate Paxlovid on the day of diagnosis, even without the approval of a physician. It is, thus, particularly difficult to initiate, follow-up, and/or evaluate the efficacy of PrEP in such a context. Another example is China with its dynamic zero COVID-19 policy. Indeed, this policy is ambitious; however, it does not favor implementation of strategies such as our proposed SARS-CoV-2 PrEP strategy as currently, (i) known cases are closely monitored, following stringent protocols, (ii) quarantine measures are largely implemented, (iii) and PCR tests are considered the gold standard. Perhaps, a PrEP strategy could be implemented if and when the prevailing COVID-19 situation in China becomes totally under control; however, the evaluation of its efficacy will remain difficult, as other associated measures aiming to reduce the exposure to SARS-CoV-2 (facemask use, decontamination measures) are ubiquitously and universally maintained.

## Conclusion

HIV PrEP has been demonstrated to be an effective infection prevention strategy, with a significantly favorable benefit-risk profile for the prevention of HIV transmission to people at high risk. Based on this model, we propose the development of SARS-CoV-2 PrEP for use in high-risk populations, including healthcare workers who can induce secondary transmission, immunodeficient individuals, and poor vaccine responders. Much progress has been made in discerning the risk factors for acquiring COVID-19, which include close contact, demographic factors, presence of certain comorbidities, environmental factors, and vaccine response. Emergent drugs with beneficial effects are paving the way for development of possible PrEP strategies which could be utilized to prevent COVID-19 infection in high-risk populations. However, several challenges exist for the development of strategies for SARS-CoV-2 PrEP, such as drug toxicity and patient safety concerns, emergence of drug resistance, and the cost of drugs. We believe that collaborative efforts at conducting comprehensive assessments for ethical considerations related to SARS-CoV-2 PrEP, the benefit-risk profiles of SARS-CoV-2 PrEP, and strategic planning of implementation of SARS-CoV-2 PrEP in selected populations should be a research priority. Based on current evidence, we consider that PrEP for COVID-19 could be a potentially useful and practical adjunct to COVID-19 vaccination to prevent SARS-CoV-2 acquisition in selected at-risk patients.

## Author contributions

JO and SZ wrote the first draft edition of the manuscript. VH and XL provided critical revisions of this manuscript. J-PR and YC conceived and designed the manuscript. All authors listed have made a substantial, direct, and intellectual contribution to the work and approved it for publication.

## Funding

This work was supported by the Joint Medical Research Project (2020GDRC010) of Chongqing Science and Technology Bureau and Chongqing Health Commission and in part by réseau SIDA and maladies infectieuses du fonds de la recherche Québec en Santé (FRQ-S). J-PR is the holder of the Louis Lowenstein Chair in Hematology and Oncology, McGill University.

## Conflict of interest

The authors declare that the research was conducted in the absence of any commercial or financial relationships that could be construed as a potential conflict of interest.

## Publisher's note

All claims expressed in this article are solely those of the authors and do not necessarily represent those of their affiliated organizations, or those of the publisher, the editors and the reviewers. Any product that may be evaluated in this article, or claim that may be made by its manufacturer, is not guaranteed or endorsed by the publisher.
